# Homeostatic Properties and Phenotypic Maturation of Murine CD4^+^ Pre-Thymic Emigrants in the Thymus

**DOI:** 10.1371/journal.pone.0056378

**Published:** 2013-02-11

**Authors:** Jie Dong, Yu Chen, Xi Xu, Rong Jin, Fei Teng, Fan Yan, Hui Tang, Pingping Li, Xiuyuan Sun, Yan Li, Hounan Wu, Yu Zhang, Qing Ge

**Affiliations:** 1 Key Laboratory of Medical Immunology, Ministry of Health, Department of Immunology, Peking University Health Science Center, Beijing, China; 2 Peking University Medical and Health Analytical Center, Peking University Health Science Center, Beijing, China; Institute of Zoology, Chinese Academy of Sciences, China

## Abstract

After a tightly regulated developmental program in the thymus, “mature” single positive (SP) thymocytes leave the thymus and enter the periphery. These newly arrived recent thymic emigrants (RTEs) are phenotypically and functionally immature, and will complete a dynamic maturation in the peripheral lymphoid organs before being licensed to be resident naïve T cells. To study the early events occurring in the RTE maturation process, we identified the phenotype of CD4^+^ pre-RTEs, a population of CD4^+^ SP thymocytes that have acquired the thymus egress capability. Compared to peripheral naïve T cells, CD4^+^ pre-RTEs displayed superior survival capability in lymphoreplete mice and faster proliferation under lymphopenic condition. The differences in Bcl2/Bim expression and/or heightened IL-7 signaling pathway may account for the pre-RTEs’ better responsiveness to homeostatic signals. Qa2, the expression of which indicates the phenotypic maturation of SPs and RTEs, was found to be upregulated in CD4^+^ pre-RTEs in thymic perivascular space. Migratory dendritic cells that surround this region contribute to Qa2 expression in pre-RTEs. The dendritic cell-driven Qa2 induction of CD4^+^ pre-RTEs is independent of MHC class II and Aire molecules.

## Introduction

Recent thymic emigrants (RTEs) comprise the population of peripheral T cells that have recently completed a tightly regulated developmental program in the thymus and entered the circulating naïve pool. Continuous production of RTEs has been shown to be critical in establishing and maintaining the diversity of the T cell repertoire, especially in those infected with certain types of viruses or who have received therapeutic lymphoablation [1], [2], [3]. RTEs are phenotypically distinct from most CD4 or CD8 single positive (SP) thymocytes and a phenotypic and functional maturation process is required before they acquire egress capability [4], [5], [6], [7], [8], [9], [10], [11], [12], [13], [14], [15], [16]. The thymic medullary microenvironment that includes both medullary thymic epithelial cells and dendritic cells (DCs) is important for this SP maturation process. RTEs are also phenotypically and functionally distinct from resident naïve T cells in the periphery [17], [18], [19], [20], [21], [22], [23], [24], [25]. Secondary lymphoid organs (SLOs) and DCs are important for the maturation process of RTEs in the periphery over a 2–3-week period [17], [26], [27]. Despite of these findings, very little is known about the molecular mechanism of this maturation process.

Multiple methods have been used to study RTEs. The commonly used one recently was RAG2p-GFP transgenic mouse model [17] that allows the identification of RTEs from unmanipulated mice. Live RTEs from secondary lymphoid organs (SLOs) can be purified from these mice and it enables the ready analysis of their phenotype, function, and migration. However, the maturation of thymic emigrants may involve multiple steps at various locations. RTEs collected from SLOs may represent cells at one of those stages that have already received some maturation signals. Thus, identification of cells that are ready to leave the thymus (termed as pre-RTEs) but are not affected by the microenvironment outside of the thymus is important to understand the early stages of RTE maturation.

We have previously resolved TCRαβ^+^CD4^+^ SP thymocytes into four subsets: SP1 (6C10^+^CD69^+^), SP2 (6C10^-^CD69^+^), SP3 (CD69^-^Qa2^-^), and SP4 (CD69^-^Qa2^+^) and proved that they define a linear, multiple-stage maturation program for the newly generated CD4^+^ SP thymocytes prior to their exportation to the periphery [4], [5]. Comparative gene expression analysis of these four subsets revealed that thymocytes at the SP4 stage are the most mature ones and acquire the thymus egress capability by expressing the highest levels of S1P_1_ and CD62L [28]. An adoptive transfer of various SP subsets directly into the thymus also supported SP4 cells as the main population that leave the thymus and enter the periphery [4]. To confirm that SP4 thymocytes are pre-RTEs that can exit the thymus and become RTEs in unmanipulated mice, we characterized in this study the phenotype of GFP^hi^CD4^+^ RTEs in adult RAG2p-GFP transgenic mice and found that they had similar phenotype as SP4 cells. In mice within 2 weeks, however, pre-RTEs had a mixed phenotype with majority of cells showing CD69^-^Qa2^-^ (a phenotype of SP3 thymocytes). Compared to mature naïve T cells, pre-RTEs showed better capabilities in survival and homeostatic proliferation. Qa2, an indicator of the phenotypic maturation of SP thymocytes and RTEs, was found to be upregulated just prior to or during the emigration of pre-RTEs. The Qa2 upregulation was driven, at least partially, by dendritic cells around the thymic perivascular space.

## Materials and Methods

### Mice

C57BL/6 congenic mice, CD45.1 and CD45.2 were purchased from Peking University Health Science Center and Vital River Lab Animal Technology Company (Beijing, China), respectively. FVB-Tg (Rag2-EGFP) 1Mnz/J mice were purchased from Jackson Laboratory (Bar Harbor, ME) and were backcrossed 10 generations onto the C57BL/6 background (termed as RAG2p-GFP in this paper). Aire^-/-^ mice were generously provided by Yangxin Fu (University of Chicago, IL) and were bred with RAG2p-GFP to generate *Aire* ^-/-^ RAG-GFP mice. B6.129S2-*H2^dlAb1-Ea^*/J mice (MHC II^-/-^) were kindly provided by Xuetao Cao (Second Military Medical University, Shanghai, China). The animals were kept in a specific pathogen-free facility at Peking University Health Science Center (Beijing, China). The experimental procedures on use and care of animals had been approved by the ethics committee of Peking University Health Science Center.

### Reagents

Anti-CD8 (3.155) was prepared from a hybridoma obtained from American Type Culture Collection (Manassas, VA). The 6C10 (SM6C10) was a kind gift from Dr. Linna Ding (National Institutes of Health) and was subsequently labeled with FITC. Anti-mouse CD127 (A7R34) and Alexa Fluor@ 647-conjugated anti-mouse Qa-2 was purchased from BioLegend (San Diego, CA). Annexin V and PI were purchased from Biosea (Beijing, China). All other antibodies used in the study were purchased from BD PharMingen (San Diego, CA). Recombinant mouse IL-7, Flt3 ligand were purchased from R&D Systems (Minneapolis, MN). LY294002 and JAK Inhibitor I were from Merck & Co. (Darmstadt, Germany).

### Flow Cytometry and Cell Sorting

Single-cell suspensions of thymocytes derived from C57BL/6 mice at 6–8 weeks of age were treated with anti-CD8 (3.155) mAb and complement (guinea pig sera) to remove CD8^+^ cells. After two cycles of killing and removal of dead cells by density centrifugation, the viable cells were stained with CD4-PerCP-Cy7, CD8-APC-Cy7 (53–6.7), CD69-PerCP-Cy5.5, 6C10-FITC, Qa2-Biotin-strepavidin-APC, CD25-PE, and NK1.1-PE and then subjected to cell sorting (FACS AriaII). SP3 (CD4^+^CD8^-^Qa2^-^CD69^-^6C10^-^CD25^-^NK1.1^-^) and SP4 (CD4^+^CD8^-^Qa2^+^CD69^-^6C10^-^CD25^-^NK1.1^-^) cells were collected. For the isolation of CD4^+^ naïve T cells, cells from lymph nodes (including axillary, mesenteric, and inguinal lymph nodes ) with a phenotype of CD4^+^CD8^-^CD44^lo^CD62L^hi^CD25^-^NK1.1^-^ (wild type mice) or GFP^-^CD4^+^CD8^-^CD44^lo^CD62L^hi^CD25^-^NK1.1^-^ (RAG2p-GFP mice) were purified. Furthermore, GFP^+^CD4^+^CD8^-^CD44^lo^CD62L^hi^CD25^-^NK1.1^-^ T cells from lymph nodes were purified as RTEs. Both naïve T cells and RTEs were collected from 6-8 weeks-old mice. The purity of these T cell populations was >97% when reanalyzed by flow cytometry.

### Generation of bone marrow-derived dendritic cells

Bone marrow cells from C57BL/6 mice were depleted of red blood cells with ammonium chloride and were plated in six-well culture plates (2–3×10^6^ cells/well) in RPMI 1640 supplemented with 10% heat-inactivated FCS and 200 ng/ml Flt3 ligand (FLDC). From day 3 to day 5, 2 ml medium containing floating cells were removed and fresh medium containing the cytokines was added. On day 8, nonaeherent and loosely adherent cells (DC) were harvested for identification and co-culture with sorted T cells.

### RTE analysis

Two methods were used to characterize RTEs. The first one involved intrathymic injection of FITC. The detailed procedures are as the following. Mice were anesthetized with 1% napental via intraperitoneal injection. An incision was made in the sternum to reveal the thymus, and approximately 10–20 µl of FITC solution (1 mg/ml) was injected into each thymic lobe with a 30-gauge needle. Control mice were injected with PBS. The chest was closed with surgical clips in the overlying skin. Twenty-four hours later, the mice were sacrificed and FITC^+^CD4^+^ T cells in the lymph nodes and spleen were collected.

The second method involved RAG2p-GFP transgenic mice. The GFP fluorescence intensity was used to determine GFP^hi^ (RTEs) and GFP^-^ (non-RTEs) cells.

To characterize the cells in the thymic perivascular space, PE-conjugated CD4 antibody was introduced into RAG2p-GFP mice via tail vein injection. Five minutes later, the mice were sacrificed and the cells with a phenotype of PE^+^GFP^hi^CD8^-^TCRβ^+^ were collected.

### *In vitro* T cell culture

Sorted CD4^+^ T cells (SP3, SP4 and naïve) were plated alone (2×10^6^/ml) or with DCs in 48- or 24-well plate at 10∶1 ratio in RPMI 1640 supplemented with 10% heat-inactivated FCS. The addition of IL-7 was at 1 ng/ml or as indicated. T cell survival was measured by staining of cells with CD4, annexin V, and PI. CFSE-labeling of T cells was used to monitor the T cell proliferation under various conditions. T cells were incubated with 2.5 mM CFSE for 5 minutes at room temperature. The cells were then washed by PBS for two times.

### Adoptive transfer experiments

Purified CD45.1^+^ naïve CD4^+^ T cells from lymph nodes (CFSE-labeled) and SP4 thymocytes were mixed at 1:1 ratio and transferred into CD45.2^+^ C57BL/6 mice via tail vein injection (2.5×10^6^ cells). An aliquot of the injected mix was stained to verify the input SP4:naïve T ratio. After 7 days, lymphocytes from blood, spleen, and lymph nodes were collected and the numbers of donor T cells were calculated by gating at CD45.1^+^CFSE^+^ (naïve) and CD45.1^+^CFSE^-^ (SP4) cells. In the experiment comparing RTEs and SP4 cells, CD45.2^+^GFP^hi^CD4^+^ T cells from lymph nodes (CFSE-labeled) and SP4 thymocytes were mixed at 1:1 ratio and transferred into CD45.1^+^ C57BL/6 mice. The persistence of donor T cells was analyzed 7 days later. When lymphopenia-induced proliferation was examined, adult lymphopenic hosts were prepared by sublethal irradiation of 8-week-old C57BL/6 mice (600 rads) one day before the adoptive transfer. The donor T cells were labeled with CFSE and analyzed 7 days (irradiated 8-week-old B6 mice) or 14 days (unirradiated 9-day-old B6 mice) after adoptive transfer.

### Quantitative RT-PCR

RNA was purified from isolated thymocytes using TRIZOL (Invitrogen) and was used to make cDNA using random primers and the Reverse Transcription System (Promega). For quantitative Real-Time PCR, iQ^TM^ SYBR® Green Supermix (Bio-Rad) was used according to the manufacturer’s instructions. Quantitative PCR was performed on an iCycler (Bio-Rad Ltd, Hemel Hempstead, England, UK), with each sample in triplicate, at an RNA equivalence of 15 ng/well. Primers for Bcl2l11 is 5′-CACAGGAGCTGCGGCGGATC-3′ and 5′-CCGCCACGTATCCTGGCTGC-3′. PCRs were performed for 40 cycles at 95°C for 30 s, 58°C for 30 s, and 72°C for 20 s. The quantification was based on ΔΔCT calculations and were normalized to GAPDH as loading controls and calibrated to SP3 levels. RT-PCR analysis was carried out on RNA from three independently isolated cell populations for each thymocyte subset and naïve T cells.

### Statistics

Data are presented as mean values ± standard deviation. Statistical significance between two groups was evaluated by two-tailed unpaired Student *t* test. Throughout the text, figures, and figure legends, the following terminology is used to denote statistical significance: *p<0.05, **p<0.01.

## Results

### CD4^+^ pre-RTEs are composed of SP4 thymocytes in adult mice and a mixture of SP3 and SP4 thymocytes in young mice

**Figure 1:**
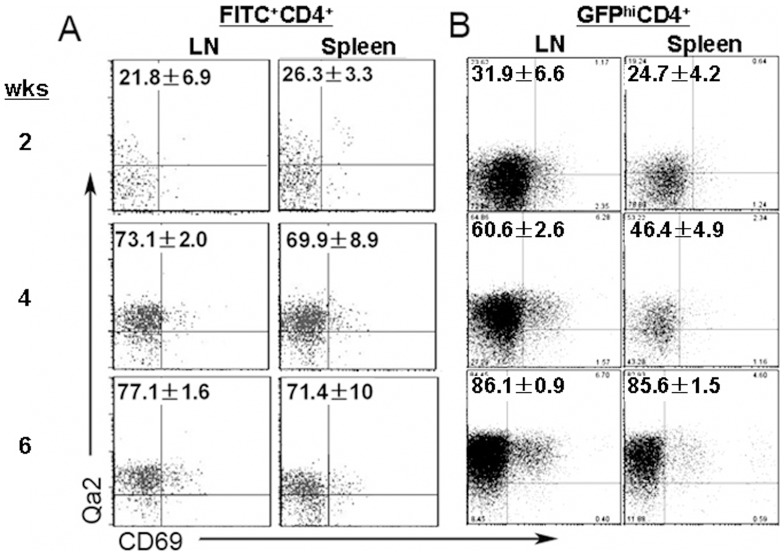
Pre-RTEs consist of SP4 thymocytes in adult mice but a mixture of SP3 and SP4 thymocytes in neonatal and young mice. A. FITC intrathymic injection of C57BL/6 mice were used to analyze the phenotype of RTEs (FITC^+^ cells) at 2, 4, and 6 weeks of age. B. RAG2p-GFP mice were also used to study RTEs (GFP^hi^ cells). The numbers in the plot represent the mean and standard deviation of the ratios of Qa2^+^CD69^-^ cells in FITC^+^ or GFP^hi^ CD4^+^CD8^-^ T cells in the lymph nodes (LN) and spleen. Three to five mice were analyzed.

CD4^+^ SP thymocytes with SP4 phenotype have been shown to express the highest levels of S1P_1_ and CD62L compared to other SP subsets. It is thus expected that SP4 thymocytes are pre-RTEs in the thymus that have acquired egress capability. However, whether these cells compose the majority of pre-RTEs have not been directly proved. Two approaches were used to examine the phenotype of emigrants that have just left the thymus. First, FITC was introduced into the mice via intrathymic injection and the phenotype of FITC^+^CD4^+^ T cells in the lymph nodes and the spleen were analyzed 24 hours later (Fig. 1A). Second, RAG2p-GFP mice were sacrificed and the phenotype of GFP^hi^CD4^+^ T cells in the periphery was examined (Fig. 1B). Boursalian et al. have shown that GFP^hi^ T cells in the periphery are RTEs that have left the thymus within a week [17]. Both methods gave very similar results that in mice with 6 weeks of age or older, more than 70% of RTEs were Qa2^+^, suggesting that the majority of pre-RTEs are SP4 thymocytes. In mice within 2 weeks of age, however, more than 70% of RTEs were Qa2^-^CD69^-^, indicating that thymocytes at the immature SP3 stage acquire egress capability and compose the main population of pre-RTEs during this period (Fig. 1A and 1B). As the appearance of SP4 cells in the thymus was not found until mice reaching 1 week of age [4], the rushing of cells with the SP3 phenotype out to the periphery at the neonatal stage may be important in establishing the peripheral T cell pool [29]. Alternatively, the SP3 thymocytes in neonatal and young mice may be different from the SP3s in adult mice. To exclude this possibility and to focus on pre-RTEs in adult animals, we used SP4 thymocytes purified from 6–8-week-old mice as pre-RTEs in the studies shown below.

### Responses of CD4^+^ pre-RTEs to homeostatic signals

**Figure 2:**
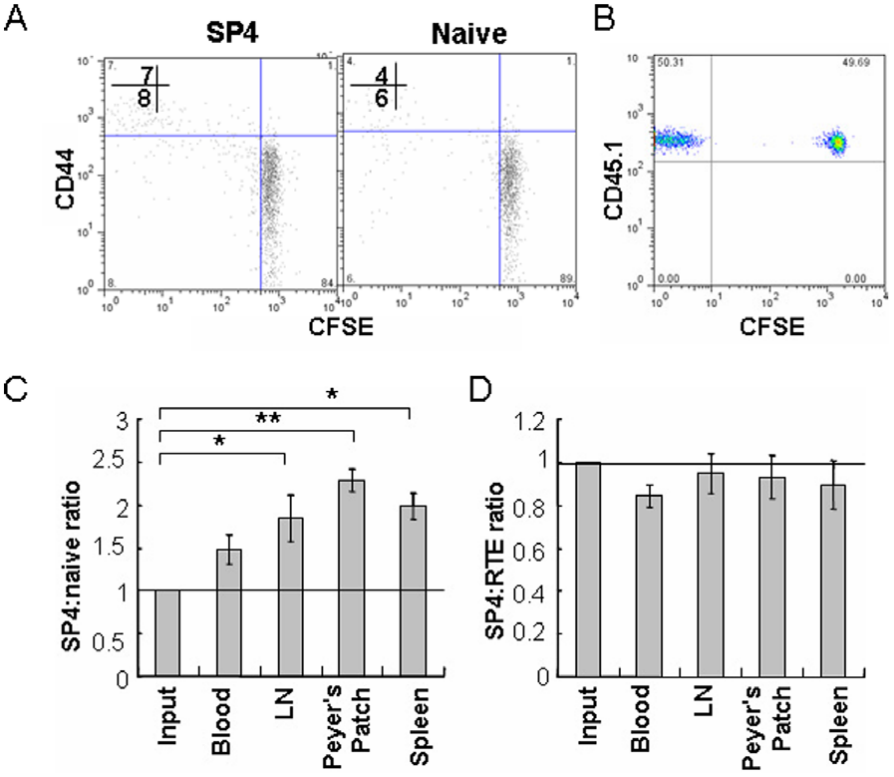
Comparison of the survival of pre-RTEs (SP4 in adult animals) and naïve T cells in mice. (A) Purified SP4 thymocytes, and CD4^+^ naïve T cells from lymph nodes of C57BL/6 mice were labeled with CFSE and adoptively transferred into unirradiated adult C57BL/6 mice. Donor T cells from lymph nodes (LN) of the hosts were analyzed for CD44 expression and CFSE dilution (proliferation) 7 days later. The numbers in the plot display the percentage of CD44^hi^ (upper) and CD44^lo^ (lower) cells that diluted CFSE. (B) Unlabeled SP4 thymocytes (pre-RTEs) and CFSE-labeled CD4^+^ naïve T cells of CD45.1 congenic mice were mixed at 1:1 ratio and were adoptively transferred into CD45.2 C57BL/6 mice. Seven days later, the mice were sacrificed and cells were harvested from peripheral blood, lymph nodes, peyer’s patch, and spleen. Donor cells collected from host lymph nodes were shown as CD45.1^+^CFSE^+^ (naïve T) and CD45.1^+^CFSE^-^ (SP4). (C) The ratio of SP4 and naïve T cells (gated as (B)) after seven days of adoptive transfer was calculated. (D) Unlabeled SP4 thymocytes and CFSE-labeled GFP^+^CD4^+^CD8^-^ lymph node cells (RTEs) from CD45.2 mice were mixed at 1:1 ratio and were transferred into CD45.1 mice. The donor cells were gated at CD45.2^+^CFSE^+^ (RTEs) and CD45.2^+^CFSE^-^ (SP4) and the ratio was analyzed 7 days later. The experiments were repeated twice and similar results were obtained. Error bars represent SEM. *P<0.05, **P<0.01, using an unpaired Student’s *t* test. N = 3-4 mice per group.

The establishment and maintenance of a diversified T cell repertoire in the periphery requires the continual egress of pre-RTEs. Thus, the ability of pre-RTEs to respond to homeostatic signals was examined. CD4^+^CD44^lo^CD62L^hi^CD25^-^ naïve T cells and GFP^hi^CD4^+^ RTEs purified from lymph nodes were used as controls. As neither SP4 thymocytes nor naïve T cells proliferated in lymphoreplete mice within 7 days (Fig. 2A), we first compared the survival of these cells in mice. SP4 thymocytes and naïve T cells from CD45.1 congenic mice were mixed at 1:1 ratio and were adoptively transferred into CD45.2 congenic mice. As shown in Fig. 2C, SP4 thymocytes persist slightly but significantly better than naïve T cells in the lymph nodes and spleens of the hosts over a 7-day period (Fig. 2B-C). Similar results were obtained by adoptively transferring SP4 thymocytes and naïve T cells separately into congenic mice (data not shown). Houston, et al. showed that naïve T cells persist better than RTEs in lymphoreplete mice [30]. It implicates that there is a survival difference between pre-RTEs and RTEs. To examine this, SP4 thymocytes and RTEs purified from CD45.2 mice were co-injected into CD45.1 congenic mice. As shown in Fig. 2D, pre-RTEs and RTEs revealed very close capability in survival.

**Figure 3:**
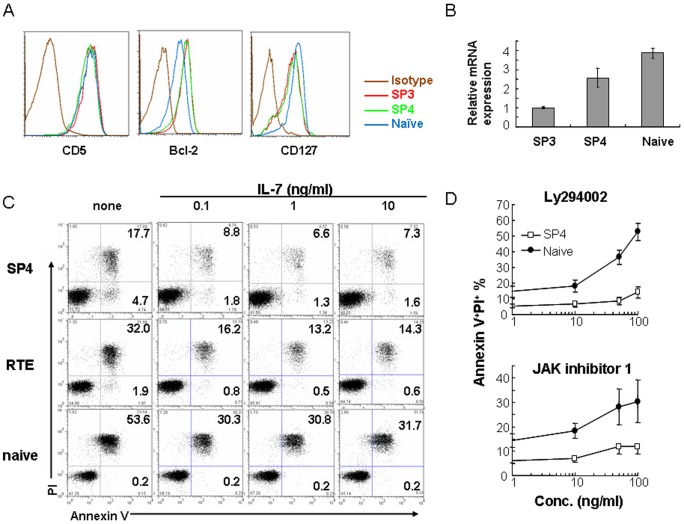
Comparison of the survival of pre-RTEs and naïve T cells in tissue culture. (A) Phenotypic comparison of freshly isolated SP4 thymocytes and CD4^+^ naïve T cells. SP3 thymocytes were used as a control. (B) Comparsion of Bim (*Bcl2l11*) expression among SP3, SP4 thymocytes, and CD4^+^ naïve T cells. Total RNAs were extracted from the three purified T cell populations. Reverse transcription and quantitative PCR was performed using Bim-specific primers. Asterisks indicate significant (*, p < 0.05; **, P<0.01) changes compared between the two subsets by unpaired Student *t* test. (C) Purified SP4 thymocytes, RTEs and CD4^+^ naïve T cells from lymph nodes of C57BL/6 mice were incubated without or with indicated concentrations of IL-7. Annexin V and PI staining of these T cells were analyzed 24 hours later. The numbers in the plot represent the percentage of Annexin V^+^PI^+^ or Annexin V^+^PI^-^ cells. The results were obtained from one of the three similar experiments. (D) Various concentrations of LY294002 and JAK inhibitor 1 were added to the T cell culture described in C. Annexin V and PI staining of these T cells were analyzed 24 hours later. The differences in the increase of Annexin V positive cells at 50 ng/ml of LY294002 and 100 ng/ml of JAK inhibitor I was significant (P<0.05) when SP4 and naïve T cells were compared by unpaired Student *t* test.

The better persistence of pre-RTEs and RTEs than naïve T cells indicates that these young T cells either have intrinsic advantage in survival or have better responses to homeostatic signals such as IL-7. Thus, we first examined the expression of antiapoptotic molecule B cell lymphoma 2 (Bcl-2) and proapoptotic molecule Bim by flow cytometry and real time PCR, respectively. Pre-RTEs displayed higher Bcl-2 and lower Bim levels as compared to peripheral naïve T cells, indicating an autonomous survival advantage of pre-RTEs (Fig. 3A and 3B). In agreement with this result, less apoptotic cells were found in pre-RTEs and RTEs cells in the absence of IL-7 *in vitro* (Fig. 3C).

The presence of IL-7 promoted the survival of all three cell populations. But again, pre-RTEs and RTEs survived better than naïve T cells even when the concentration of IL-7 was as high as 10 ng/ml (Fig. 3C). Compared to naïve T cells, pre-RTEs were less sensitive to inhibitors that block IL-7 signaling pathways, such as phosphoinositide 3-kinase (PI3K) inhibitor Ly294002 and Jak kinase inhibitor 1 (Fig. 3D). These differences in IL-7 signaling pathways suggest that pre-RTEs have better survival responses to IL-7 than naïve T cells. The only exception is the IL-7 receptor (IL-7)α expression as lower level of this receptor was found in pre-RTEs (Fig. 3A). It is possible that the downregulation of IL-7Rα in pre-RTEs is simply a result of higher sensitivity in IL-7 signaling. No differences were found in CD5 expression between pre-RTEs and naïve T cells, suggesting that TCR signaling may be similar between these two populations (Fig. 3A).

**Figure 4:**
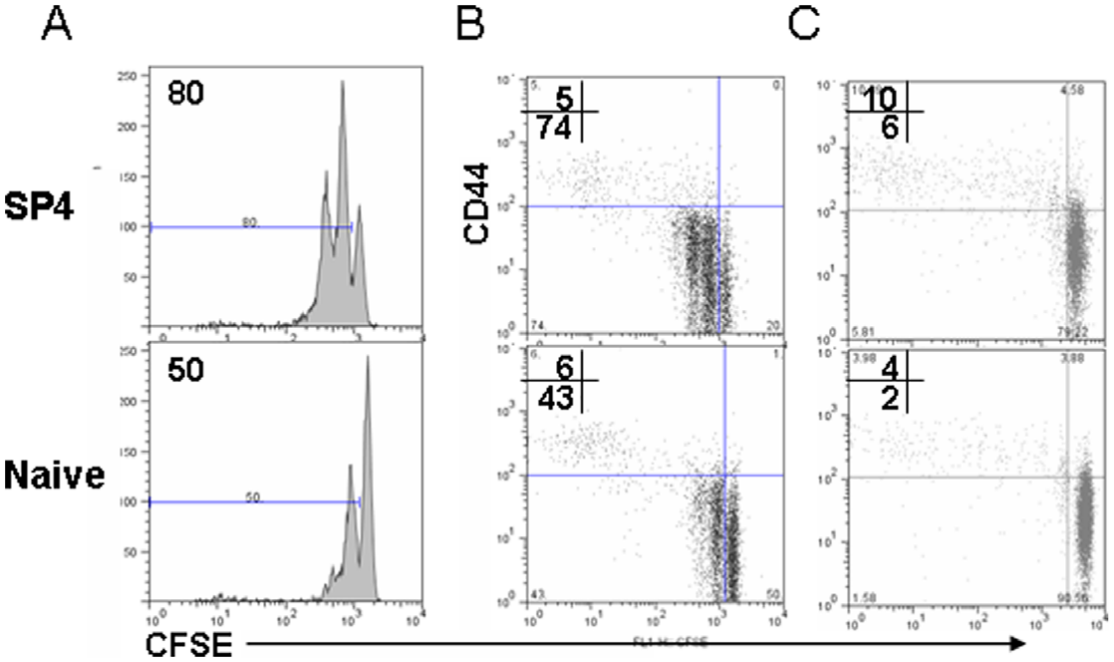
Comparison of lymphopenia-induced proliferation of pre-RTEs and naïve T cells. Purified SP4 thymocytes, and CD4^+^ naïve T cells from lymph nodes of C57BL/6 mice were labeled with CFSE and adoptively transferred into sub-lethally irradiated (600 rads) adult C57BL/6 mice (A and B) or unirradiated 9-day old C57BL/6 mice (C). Donor T cells from lymph nodes (LN) of the hosts were analyzed for CD44 expression and CFSE dilution (proliferation) 4 days (irradiated mice) and 14 days (unirradiated young mice) later. The numbers in the plot display the percentage of cells diluted CFSE (A) or CD44^hi^ (upper) and CD44^lo^ (lower) cells that diluted CFSE (B-D). The experiments were repeated for 3 times.

We next compared the capability of lymphopenia-induced proliferation between pre-RTEs and lymph node-resident CD4^+^ naïve T cells. Both SP3 and SP4 pre-RTEs proliferated significantly faster than naïve T cells in the adult mice that received sub-lethal dose of irradiation (Fig. 4A and 4B). Similar results were obtained in the hosts of nine-day old young mice in which the peripheral T cell pool was not yet completely filled (Fig. 4C). This is consistent with the findings by Houston, et al. that RTEs proliferated faster than naïve T cells in lymphopenic condition [30].

**Figure 5:**
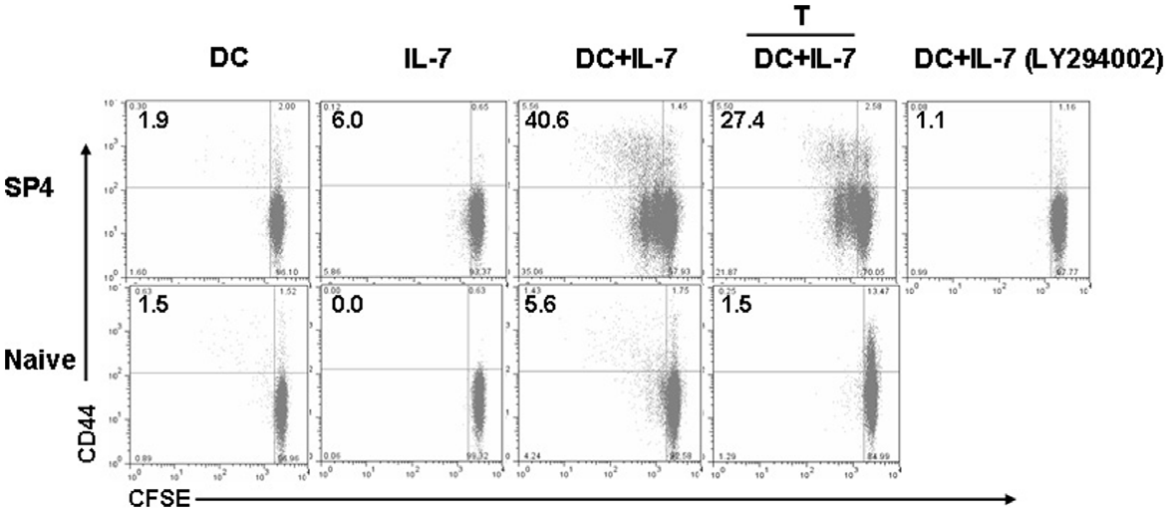
Comparison of the proliferation of pre-RTEs and naïve T cells in T-DC coculture system. Purified pre-RTEs (SP4 thymocytes) and naïve CD4^+^ T cells were labeled with CFSE and cultured with FLDCs at 10:1 ratio. The proliferation and CD44 upregulation were analyzed 4 days later. The fourth column displayed the T-DC coculture in the transwell system. T cells and DCs were separated by 0.4 µm transwell membrane. In the last column, Ly294002 was added to the T-DC coculture at 10 ng/ml. The numbers in the plot indicate the percentage of cells that diluted CFSE. The experiment was repeated for at least 3 times.

As dendritic cells (DCs) are essential in driving lymphopenia-induced naïve T cell proliferation, we tested whether pre-RTEs respond to DC-driven proliferation differently *in vitro*. Pre-RTEs and CD4^+^ naïve T cells were cultured in the presence of DCs and IL-7. Within 4 days, very little cells diluted the CFSE dye in naïve T cells whereas at least two cycles of cell division could be found in SP4 thymocytes (Fig. 5). The proliferation of pre-RTEs was not completely blocked by separating DCs and T cells with a transwell membrane, suggesting that cytokines produced by DCs play a role in driving T cell proliferation in this system. Inhibitors blocking PI3K could completely suppress the proliferation (Fig. 5).

### Dendritic cells promote the phenotypic maturation of CD4^+^ pre-RTEs

**Figure 6:**
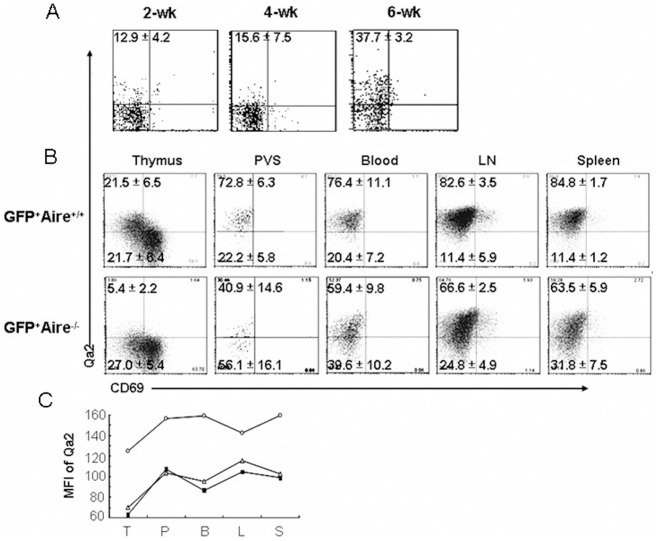
pre-RTEs in the perivascular space upregulate their Qa2 expression. (A) Phenotype of RTEs in Aire^-/-^ mice. FITC intrathymic injection of Aire^-/-^ mice was used to analyze the phenotype of RTEs (FITC^+^CD4^+^CD8^-^ cells) at 2, 4, and 6 weeks of age. The data from the littermate Aire^+/+^ control mice were shown in Fig. 1A. (B) Phenotype of GFP^hi^CD4^+^CD8^-^ T cells in RAG2p-GFP and RAG-GFP-Aire^-/-^ mice. GFP^hi^CD4^+^CD8^-^ T cells from thymus, peripheral blood, lymph nodes, spleen were gated and the expressions of Qa2 and CD69 were analyzed. T cells in the PVS were analyzed by gating at GFP^hi^PE^+^CD8^-^TCRβ^+^ thymocytes 5 minutes after tail vein injection of PE-conjugated CD4 antibody into mice. The numbers in the plot represent the mean and standard deviation of the ratios of Qa2^+^CD69^-^ cells in FITC^+^, Qa2^+^CD69^-^ cells or Qa2^-^CD69^-^ cells in GFP^hi^CD4^+^CD8^-^ T cells. Three to five mice were analyzed. (C) Mean fluorescence intensity of Qa2 in GFP^hi^CD4^+^CD8^-^ T cells from various places was compared. T: thymus (GFP^hi^CD4^+^CD8^-^Qa2^+^CD69^-^ cells); P: PVS; B: blood; L: lymph nodes; S: spleen. The data represent the analysis of three mice.

The expression level of Qa2 is an indicator for the phenotypic maturation of SP thymocytes as well as RTEs. In *Aire* ^-/-^ mice, very little Qa2^+^ thymocytes (SP4) could be found in the thymus. The RTEs in these mice were thus expected to have an immature SP3 phenotype. In an attempt to study whether the exportation of SP3 cells in adult mice results in differences in maturation of RTEs, we unexpectedly found that almost 40% of RTEs (FITC^+^ T cells in the periphery 24 hours after FITC intrathymic injection or GFP^hi^CD4^+^ T cells in the periphery of RAG2p-GFP-*Aire* ^-/-^ mice) were Qa2 positive (Fig 6A–B). This result suggests that some SP3 thymocytes may start to express this molecule during their thymus egress or during the time when RTEs enter the SLOs. To distinguish these two possibilities, PE-conjugated anti-CD4 antibody was introduced into RAG2p-GFP-*Aire* ^-/-^ mice via tail vein injection. The PE^+^TCRβ^+^ thymocytes were analyzed 5 minutes later. Zachariah et al. have shown that cells in the perivascular space of the thymus (PVS, a main region where pre-RTEs exit the thymus) could be labeled using this method [31]. Similar to the results obtained from the lymph nodes and spleen, about 40% of PE-labeled GFP^hi^ T cells in the thymic PVS of RAG2p-GFP-*Aire* ^-/-^ mice were Qa2 positive (Fig. 6B). In addition, GFP^hi^ T cells in the thymic PVS, peripheral blood, lymph nodes, and spleen express similar levels of Qa2 whereas GFP^hi^ SP4 thymocytes express much lower level of Qa2 (Fig. 6C). This indicates that Qa2 upregulation was initiated around the thymic PVS region and did not significantly change after RTEs leaving the thymus and staying in the SLOs for about a week.

**Figure 7:**
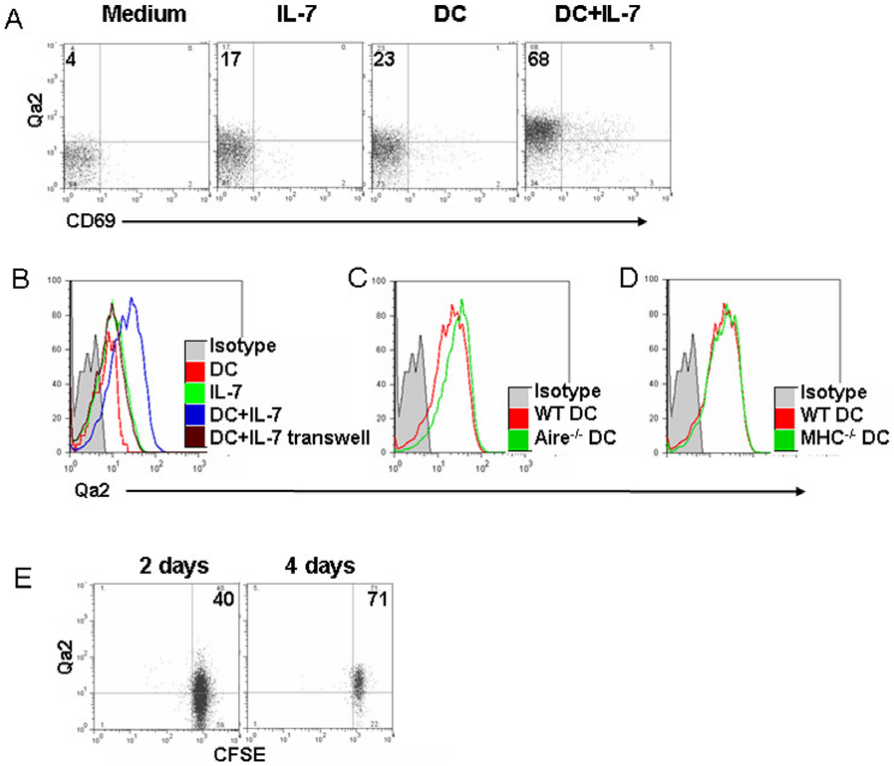
Dendritic cells promote the Qa2 upregulation of SP3 pre-RTEs. Purified SP3 thymocytes were cultured with IL-7 and FLDCs derived from C57BL/6 mice (A, B, E), and *Aire* ^-/-^ mice (C), and MHC class II^-/-^ mice (D) for 4 days. The expression of Qa2 (B–D) and in some cases, Qa2 and CD69 (A) of live T cells were analyzed. (E) SP3 thymocytes upregulate their Qa2 expression in lymphoreplete mice. CFSE-labeled SP3 thymocytes were adoptively transferred into C57BL/6 mice. Qa2 expression was analyzed in CFSE^+^ T cells in the lymph nodes and spleen at 2 and 4 days post-transfer. The numbers show the percentage of Qa2^+^ cells. The experiments have been repeated for 3 times.

We have previously shown that thymic epithelial cells were required for Qa2 expression in the thymus that indicates the SP3-to-SP4 transition [5]. However, thymic epithelial cells purified from *Aire* ^-/-^ mice failed to induce Qa2 upregulation in wild type SP3 cells *in vitro* (Yu, S., Zhang, Y., manuscript in preparation). Thus, stromal cells other than epithelial cells may contribute to the induction of Qa2 expression in the thymic PVS in *Aire* ^-/-^ mice. Dendritic cell, including SIRPα^-^ resident conventional DC (cDC), migratory SIRPα^+^ cDC, and migratory plasmacytoid DC (pDCs), is a type of thymic stromal cells that plays an important role in negative selection [32], [33], [34], [35], [36]. We thus examined whether DCs promote the phenotypic maturation of pre-RTEs. As only Flt3 ligand (Flt3L)-induced DCs (FLDCs) have been shown to enter the thymus via PVS region [37], [38], SP3 thymocytes (CD69^-^Qa2^-^) were purified from B6 mice and were cultured with FLDCs *in vitro*. To promote the survival of thymocytes, 1 ng/ml of IL-7 was added in the co-culture system. About 20% of SP3 cells upregulated their Qa2 when T cells were cultured with IL-7 alone, DCs alone, or cultured in the transwell with IL-7 and DCs (T cells in the upper well and DCs in the lower well) (Fig. 7A–B). However, more than 60% of T cells expressed Qa2 when they were cultured with IL-7 and DCs in the same well (Fig. 7A), suggesting that the cellular interaction between T and DCs was essential in this process. FLDCs deficient in *Aire* were further examined and similar levels of Qa2 expression were found in the culture with *Aire* ^-/-^ or wild type DCs (Fig. 7C).

To examine whether TCR-MHC interaction is critical in the phenotypic maturation of pre-RTEs, FLDCs from MHC class II-deficient mice were used in the T-DC coculture system and no differences in Qa2 expression were found between the culture with knockout DCs and wild type DCs (Fig. 7D). Cell division is not required as Qa2 upregulation was found in the CFSE^hi^ non-proliferating cells (data not shown). Fink et al. reported that peripheral lymphoid organs and DCs, but not MHC II, are required for Qa2 upregulation of RTEs [26], [27]. Our results further suggest that Qa2 expression of pre-RTEs in the thymus also depends on DCs, but not depends on MHC II expression on DCs. When we introduced SP3 pre-RTEs into lymphoreplete mice via tail vein injection, the kinetics and levels of Qa2 expression in mice was very similar to that in T-DC coculture (Fig 7E), implicating that similar DCs, probably migratory DCs, may contribute to the phenotypic maturation of pre-RTEs in the thymus and RTEs in the periphery.

## Discussion

SP thymocytes and RTEs have been shown to be heterogeneous and undergo maturation before becoming mature resident naïve T cells. Such a maturation process is dynamic and can be affected by different microenvironments of the thymus, SLOs, and even peripheral tissues [26]. Thus, choosing which population, SP thymocytes, RTE wannabes (pre-RTEs) in the thymus, or RTEs in the lymph nodes, to study the maturation process may be important and may lead to different findings reflecting different stages of maturation. In the present study, we identified thymic CD4^+^ pre-RTEs in adult and young mice. We then focused on the ones in adult animals (SP4 thymocytes) and investigated their phenotypic and homeostatic properties. Compared to mature naïve T cells purified from lymph nodes, CD4^+^ pre-RTEs showed superior capabilities to survive in the periphery and proliferate in lymphopenic hosts. This homeostatic advantage of pre-RTEs may lead to preferential incorporation of newly generated T cells with new repertoire diversity into peripheral T cell pool. Such a diversified TCR repertoire is essential in the immunity against neoantigens [3], [39], [40], [41], [42]. In addition, the Qa2 upregulation of CD4^+^ pre-RTEs induced by migratory DCs around thymic PVS indicates that at least the phenotypic maturation of RTEs is likely initiated by DCs in the thymus. As DCs have been shown to be critical in the maturation of RTEs in the periphery [26], [27], it further implies that similar DCs may provide similar maturation signals to pre-RTEs in the thymus as well as RTEs in the periphery.

Using a thymus transplantation model, Berzins et al. found that RTEs may have different responses to homeostatic signals within a 3-week period [43]. In agreement with this finding, our data provide direct evidence that both pre-RTEs and RTEs persist better than peripheral naïve T cells in lymphoreplete mice and proliferate faster than naïve T cells in lymphopenic hosts. A slightly faster lymphopenia-induced proliferation of RTEs was also found by Houston et al. [30]. These results suggest that pre-RTEs and RTEs are both capable of filling the niche in the periphery. However, Houston showed that RTEs persist less well than naïve T cells in the lymph nodes and spleen of lymphoreplete mice. This is in contrast to our results that pre-RTEs and RTEs persist similarly to or even slightly better than naïve T cells in SLOs. There are several possible explanations for this disparity. First, RTEs and peripheral naïve T cells were purified from age-matched (6–8 weeks old) mice in our but not in Houston’s experiments. The latter group used RTEs from 5-week-old donors but naïve T cells from donors older than 12 weeks old [30]. The naïve T cells from older mice may persist longer in the periphery [44], [45]. Second, we only examined T cell survival in a short 1-week period. It is possible that RTEs decline faster than naïve T cells at a later time point.

Interestingly, a significantly better survival of CD4^+^ pre-RTEs and RTEs than naïve T cells was shown in SLOs. The balance of Bcl2/Bim and the heightened IL-7 signaling both contribute to the better persistence of pre-RTEs. A similarly enhanced response to IL-7 was also found in neonatal RTEs [25]. What drives these molecular differences between pre-RTEs and naïve T cells is not clear. It may be traced back to positive selection as it was thought positive selection-derived upregulation of early growth response protein 1 (Egr1) contributed to the survival of CD4^+^ pre-RTEs in the periphery for at least 4 days in the absence of MHC class II and IL-7 [46]. It remains to be examined whether Egr1 could affect the signaling molecules downstream of IL-7R. Nevertheless, the survival advantage of pre-RTEs may facilitate their filling and/or replacing the aging peripheral T cell pool. Without RTEs, such as in thymectomized mice and age-related thymic involution, T cells persist longer in the periphery and accumulate defects in their functions [44], [45].

Perivascular space in the thymus is a region or a “gate” where T cells use to leave or return to the thymus [31], [47]. Antigen presenting cells from the peripheral blood such as dendritic cells, macrophages, and even B cells could enter the thymus via PVS, providing antigens, adhesion molecules, and cytokines to the immature thymocytes. Maturing thymocytes may also receive signals from resident cells in or around the PVS. For instance, pericytes in the PVS were involved in the regulation of thymocyte egress by producing S1P [31]. Thus, PVS or regions surround it could be an important place in the maturation of pre-RTEs. Indeed, CD4^+^ T cells in the thymic PVS expressed similar level of Qa2 as peripheral RTEs that had left the thymus within a week but much higher level of Qa2 than the most mature SP subset (SP4). Two possibilities may account for this phenomenon. First, CD4^+^ pre-RTEs were induced by unknown factors in the thymus to upregulate Qa2 right before their entry into the PVS. Second, a small population of Qa2^hi^ cells from pre-RTEs acquired better capability to enter the PVS and subsequently exit the thymus. Our results support the first possibility. In *Aire^-/-^* mice that have an SP3-to-SP4 developmental blockage and very little Qa2 expression in CD4^+^ SP, nearly half of the CD4^+^ SPs in the thymic PVS were Qa2^+^. DCs are partially responsible for this Qa2 up-regulation around the PVS region as thymic epithelial cells from *Aire^-/-^* mice do not induce Qa2 upregulation in wild type SP3 cells (Yu, S., Zhang, Y., manuscript in preparation). DCs may also contribute to the upregulation of Qa2 found in RTE “wannabes” when egress was blocked by S1P mimics [26]. In addition, the induction of Qa2 does not require TCR-MHC interaction between pre-RTEs and DCs, such a DC-promoted phenotypic maturation of pre-RTEs within the thymus is reminiscent of DC-driven RTE maturation in the the SLOs [26], [27]. This suggests that the maturation of pre-RTEs and RTEs may both require DCs and may even be connected by similar population of migratory DCs that enter the PVS and circulate through the SLOs. As DCs may bring more self antigen to the maturing pre-RTEs and RTEs, this interaction of young T cells with DCs in the thymus and SLOs may ensure a more complete elimination or control of self-responsive RTEs.

Collectively, our findings suggest that CD4^+^ pre-RTEs have intrinsic advantages over “old” peripheral naïve T cells in the survival and lymphopenia-induced proliferation, enabling them to play an essential role in maintaining a diverse T cell repertoire in the periphery. The phenotypic maturation program of thymic emigrants starts in or around the thymic perivascular region, and is driven by migratory DCs. The significance of this DC-mediated pre-RTE maturation is currently under investigation. It can be a continuous process of self-responsiveness control or/and better fitness to the peripheral environment.
